# Vitamin D accelerates clinical recovery from tuberculosis: results of the SUCCINCT Study [Supplementary Cholecalciferol in recovery from tuberculosis]. A randomized, placebo-controlled, clinical trial of vitamin D supplementation in patients with pulmonary tuberculosis’

**DOI:** 10.1186/1471-2334-13-22

**Published:** 2013-01-19

**Authors:** Nawal Salahuddin, Farheen Ali, Zahra Hasan, Nisar Rao, Masooma Aqeel, Faisal Mahmood

**Affiliations:** 1 Consultant Department of Adult Critical Care Medicine, King Faisal Specialist Hospital & Research Centre, Riyadh, Saudi Arabia; 2 Section of Infectious Disease, Department of Medicine, Aga Khan University, Karachi, Pakistan; 3 Department of Pathology and Microbiology, Aga Khan University, Karachi, Pakistan; 4 Department of Pulmonology, Ojha Institute of Chest Diseases, Karachi, Pakistan; 5 Section of Pulmonary and Critical Care Medicine, Department of Medicine, Aga Khan University, Karachi, Pakistan; 6 Section of Infectious Disease, Department of Medicine, Aga Khan University, Karachi, Pakistan

## Abstract

**Background:**

Vitamin D enhances host protective immune responses to *Mycobacterium tuberculosis* by suppressing Interferon-gamma (IFN-g) and reducing disease associated inflammation in the host. The objectives of this study were to determine whether vitamin D supplementation to patients with tuberculosis (TB) could influence recovery.

**Methods:**

Two hundred and fifty nine patients with pulmonary TB were randomized to receive either 600,000 IU of Intramuscular vitamin D_3_ or placebo for 2 doses. Assessments were performed at 4, 8 and 12 weeks. Early secreted and T cell activated 6 kDa (ESAT6) and *Mycobacterium tuberculosis* sonicate (MTBs) antigen induced whole blood stimulated IFN-g responses were measured at 0 and 12 weeks. Statistical comparisons between outcome variables at 0 and 12 weeks were performed using Student’s *t*-test and Chi^2^ tests.

**Results:**

After 12 weeks, the vitamin D supplemented arm demonstrated significantly *greater* mean weight gain (kg) + 3.75, (3.16 – 4.34) versus + 2.61 (95% CI 1.99 – 3.23) *p* 0.009 and *lesser* residual disease by chest radiograph; number of zones involved 1.35 v/s 1.82 *p* 0.004 (95% CI 0.15, 0.79) and 50% or greater reduction in cavity size 106 (89.8%) v/s 111 (94.8%), *p* 0.035. Vitamin D supplementation led to significant increase in MTBs-induced IFN-g secretion in patients with baseline ‘Deficient’ 25-hydroxyvitamin D serum levels (*p* 0.021).

**Conclusions:**

Supplementation with high doses of vitamin D accelerated clinical, radiographic improvement in all TB patients and increased host immune activation in patients with baseline ‘Deficient’ serum vitamin D levels. These results suggest a therapeutic role for vitamin D in the treatment of TB.

**Trial registration:**

ClinicalTrials.gov; No. *NCT01130311*; URL: *clinicaltrials.gov*

## Background

Vitamin D is now known to be essential to *Mycobacterium tuberculosis* (MTB) containment and killing through activation of 25-hydroxyvitamin D receptors (VDRs) present on all immune cells. Stimulation of Toll-like receptors (TLRs) on monocytes and macrophages by MTB antigens leads to an up-regulation of VDRs. Binding of 1,25(OH)_2_ D_3_ activates VDRs and induces cathelicidin-mediated killing of Mycobacteria [1,2]. Susceptibility to tuberculosis (TB) and risk of progression from infection to disease, tends to occur more often in patients with low 25- hydroxyvitamin D levels [3-5] while, close contacts of TB patients display a 5-fold increased risk of contracting TB with each relative 1–log decrement in 25- hydroxyvitamin D level [6]. Historically cod liver oil and sunshine, both excellent sources of 25-hydroxyvitamin D, were used for the treatment of TB. More recently, single doses of 25-hydroxyvitamin D have been demonstrated to enhance mycobacterial killing [7]. However two recent Vitamin D supplementation trials failed to demonstrate any benefit [8,9] possibly due to the low doses used. We hypothesized that supplementation with *therapeutic doses* of vitamin D in patients with active TB, may improve outcomes. Our study objectives were to determine the effects of vitamin D supplementation on clinical indicators and immune responses.

## Methods

### Study design

This was a randomized double blinded, multi-centre, placebo-controlled clinical trial. The study was approved by the institutional review boards of the participating centres; Aga Khan University Hospital (ERC approval no. 1238-Med/ERC-09) and Ojha Institute of Chest Diseases, Dow University Hospital (IRB-94/DUHS-09) and is listed on clinicaltrials.gov (NCT01130311). The trial was funded through a grant obtained from the Aga Khan University Research Council (URC grant No. 0000058579). The study was conducted from October 2009 to July 2010 with enrolment of the first participant in October 2009 and last participant in April 2010. The protocol was made available at clinicaltrials.gov in May 2010. All patients provided written, informed consent prior to participation. Consecutive adult patients (≥16 years) with smear positive, active pulmonary TB diagnosed within one week and enrolled at outpatient TB clinics were included. Based on clinical history taking and clinical records, patients with extra- pulmonary TB, human immunodeficiency virus (HIV) infection, hepatic disease, renal failure, malignancy, diabetes mellitus, pregnancy, sarcoidosis, hyperparathyroidism or those taking any corticosteroids, immunosuppressive agents, thiazide diuretics or drugs known to interfere with vitamin D levels (phenytoin, phenobarbital, carbamazepine, theophylline) were excluded from the study.

### Primary and secondary outcome variables

The primary outcome variables were differences in weight gained and resolution of chest radiograph abnormalities. Secondary outcomes were differences in whole blood cell antigen-stimulated Interferon-gamma(IFN-g) responses, differences in sputum conversion rates and improvements in the TB score. An ad hoc analysis looked at the differences in the cytokine response, and clinical recovery based on differences in baseline vitamin D status.

### Measurements

Baseline clinical data, chest radiographs, sputum and blood samples for 25 hydroxyvitamin D assay and cytokine analysis were collected. The randomization sequence was generated by the Dept. of Pharmacy, Aga Khan University, who were responsible for dispensing the study drug/placebo. The study co-ordinator and physician available at the study site enrolled participants who were eligible. All patients continued to receive Directly Observed Therapy (DOTS) with 2 months of 4 antituberculous drugs [Isoniazid, Rifampicin, Ethambutol and Pyrazinamide] followed by 6 months of Isoniazid and Ethambutol. Eligible patients were screened, enrolled and randomized by a computer-generated stratified, random assignments list (block randomization). The 2 study arms were either administered 600,000 IU of intramuscular vitamin D_3_ (cholecalciferol) for 2 doses one month apart or an equivalent volume of normal saline matched for colour. Doses were given at enrolment and repeated 4 weeks from baseline. This dosage formulation of vitamin D is as recommended for vitamin D supplementation by the Pakistan Endocrine Society [*personal communication*]. The patient, primary physicians, investigator physicians, study coordinator, site assistants were blinded to the treatment allocation.

Clinical assessments and sputum microscopic examinations were performed at all visits (0, 4, 8 and 12 weeks of therapy). Chest radiographs and blood samples for cytokine assays were obtained at 0 and 12 weeks. Clinical examination was used to calculate a TB score [10] for all visits. The TB score is a validated assessment tool developed to objectively measure change in the clinical status of TB patients. Its components include self-reported symptoms (cough, shortness of breath, night sweats, chest pain, haemoptysis), clinical signs (tachycardia, pallor, fever, auscultatory findings) body mass index (BMI) and mid-upper arm circumference (MUAC). Mid Upper Arm Circumference (MUAC) was recorded to an accuracy of 0.5 cm at the mid-point of the acromion and the olecranon process over the biceps muscle of the non-dominant arm, using a non-stretchable measuring tape. Height was recorded in metres, and weight in kilograms using a standard weighing machine at all visits. Body Mass Index (BMI) = weight (kg)/height (m^2^). The TB score achieved can range from 0–13. TB scores were further divided into 3 severity classes; Severity Class I (TB score 0 to 5), Class II (TB score 6 – 7) and Class III (TB score ≥ 8). Baseline 25 hydroxyvitamin D levels were measured and divided into ‘Optimal’ > 30 ng/ml, ‘Insufficient’ 20–30 ng/ml and ‘Deficient’ < 20 ng/ml.

Chest X-rays were interpreted independently by 2 consultant pulmonologists who were blinded to the treatment allocation process. Three separate methods of disease categorization were employed. This included classification into ‘minimally’, ‘moderately’ and far advanced categories of radiographic infiltrates [11,12]. Secondly, cavity size and change in size from 0 to 12 weeks was recorded. Thirdly, the bilateral lung fields were divided in to 3 zones (6 total) and disease extent was recorded as ‘Zone involvement’ (Table 1).

**Table 1:** Baseline characteristics in the vitamin D and placebo study arms (N = 259)

**Baseline characteristic**	**Randomization**	
	**Drug intervention (n = 132)**	**Placebo intervention (n = 127)**
Age (years), Mean ± SD	27.8 ± 13.2	28.3 ± 14.1
Gender, Men	71 (53.8)	70 (55.1)
Weight (kg), Mean ± SD	45.2 ± 7.6	45.6 ± 9.0
BMI, Mean (range)	17.2 (11–25)	17.3 (11–27)
MUAC (cm), (range)	21.2 (14–30)	21.1 (15–32)
Disease Symptom(s)present		
Cough	127 (96.2)	125 (98.4)
Haemoptysis	30 (22.7)	34 (26.8)
Dyspnoea	84 (63.6)	85 (66.9)
Chest pain	84 (63.6)	84 (66.1)
Night sweats	69 (52.3)	55 (43.3)
Pale conjunctiva	70 (53)	67 (52.8)
Tachycardia	87 (65.9)	86 (67.7)
Fever	49 (37.1)	68 (53.5)
Crepitations	50 (37.9)	38 (29.9)
Rhonchi	19 (14.4)	21 (16.5)
Reduced breath sounds	26 (19.7)	20 (15.7)
TB score, Mean ± SD, 95 CI	6.68 ± 2.04, 6.3-7.03	6.85 ± 2.50, 6.4-7.29
Distribution by Severity Class/TB score*		
Class I/0 – 5	32 (24.2)	40 (31.5)
Class II/6 – 7	51 (38.6)	41 (32.3)
Class III/≥ 8	49 (37.1)	46 (36.2)
Serum-25-(OH)D_3_ levels; Mean, (SD)	20.58 ± 8.51	22.87 ± 10.33
25(OH)D_3_ >30 ng/ml (Optimal)	18 (13.6)	25 (19.7)
25(OH)D_3_ 20-30 ng/ml (Insufficient)	46 (34.8)	54 (42.5)
25(OH)D_3_ < 20 ng/ml (Deficient)	70 (53)	51(41)
Chest X-Ray Classification^1^		
Minimally Advanced disease	10 (7.6)	13 (10.2)
Moderately Advanced disease	77 (58.3)	62 (48.8)
Far Advanced disease	45 (34.1)	52 (40.9)
No Cavity	7 (5.3)	4 (3.1)
Cavity size < 4 cm	60 (45.5)	59 (46.5)
Cavity size ≥ 4 cm	65 (49.2)	64 (50.4)
No. of zones involved ^3^, Mean ± SD	3.61 ± 1.40	3.64 ± 1.48
Sputum microbial load (microscopy)^2^		
Scant, 1–9 AFB/100 fields	4 (3.0)	2 (1.6)
+1, 10–99 AFB/100 fields,	36 (27.3)	34 (26.8)
+2, 1–10 AFB/50 fields,	25 (18.9)	23 (18.1)
+3, >10 AFB/20 fields,	67 (50.8)	68 (53.5)
*IFN-g levels* (pg/ml) mean ± SD		
Unstimulated levels	0.2 ± 2.6	0 ±0
ESAT6-stimulated	413 ±977	303 ±804
MTBs-stimulated	2826 ±1391	2858 ±1337

*MUAC* Mid Upper Arm Circumference; *BMI* Body Mass Index; s-25-(OH)D_3_ = serum 25- hydroxyvitamin D_3_.* Severity Class (TB score) [10];^1^Chest X ray classification: Each hemi-lung was divided into 3 zones (total 6 lung zones): active parenchymal and cavitary disease, exclusive of old fibrotic scarring, recorded as ‘zone involvement’ [11,12]. ^2^Diagnostic Standards and Classification of Tuberculosis, National Tuberculosis Association of the USA, 1961. Data are presented here as number (%), unless stated otherwise.

At each follow up visit patients were asked about symptoms of hypercalcemia i.e. nausea, vomiting, abdominal pain, confusion or renal colic. Calcium (and albumin in patients with BMI < 18) was measured at 12 weeks from enrolment.

### Whole blood assay and IFN-g measurements

Recombinant antigen early secreted and activated target (ESAT) -6 kDa and *M. tuberculosis* H37Rv whole sonicate (MTBs) were provided through the NIH tuberculosis vaccine testing and reagent material contract (NO1-A1-40091) awarded to Colorado State University, USA. Mycobacterial antigen stimulated responses in study subjects of the placebo group (n = 127) and vitamin D intervention group (n = 132) were tested at the time of recruitment prior to any anti-tuberculosis treatment or supplementation and subsequently after treatment (weeks 12 of inclusion). Diluted whole blood cells were stimulated with 5 mcg/ml ESAT6 and 10 mcg/ml MTBs and IFN-g was measured in cell culture supernatants collected at 6 days post-stimulation as described previously [13]. All samples were set up in replicates. Samples were centrifuged to collect any cellular debris, aliquoted and stored at −70 C until tested.

IFN-g was detected in cellular supernatants by using standards and ELISA reagents obtained from Endogen (Rockford, IL, USA). Cytokines were measured using a sandwich ELISA technique according to the manufacturer’s instructions and as reported previously [13]. Recombinant human cytokine was used to obtain a dose response curve with a range of detection from 3.9-1000 pg/ml.

### Biochemical assays

Serum 25-hydroxy25-hydroxyvitamin D3 was measured using the **e**lectro**c**hemi**l**uminescence **i**mmuno**a**ssay “ECLIA” (Roche Diagnostics GmbH, D-68298 Mannheim). Serum calcium and albumin was measured by colorimetric assay (Roche Diagnostics GmbH, D-68298 Mannheim) and colorimetric determination of serum albumin using bromocresol green at pH 4.20 (Merck, Pakistan).

Clinical assessments and sputum microscopic examinations were performed at 0, 4, 8 and 12 weeks of therapy. Chest radiographs and blood samples for cytokine assays were obtained at 0 and 12 weeks. At each follow up visit patients were asked about symptoms of hypercalcemia i.e. nausea, vomiting, abdominal pain, confusion or renal colic. Serum 25-hydroxy 25-hydroxyvitamin D_3_, calcium (and albumin in patients with BMI < 18) were measured at 12 weeks from enrolment. For the sample size calculation, we hypothesized that vitamin D supplementation could result in a weight gain equal to or greater than 10% of the mean weight gain in the placebo group [8] and a 15% difference in radiographic improvement between the 2 groups. We estimated we would need 125 patients in each arm to reject the null hypothesis with a power of 80 percent and a 5% level of significance. We estimated a 15% risk of hypercalcemia after supplementation in the 25-hydroxyvitamin D arm, based on a previous study by Wejse et al. [9] and therefore estimated we would need 60 patients in each group for a power of 80% and 5% level of significance. The primary outcome variable was differences in weight gained and resolution of chest radiographic abnormalities. Secondary outcomes were differences in IFN-g responses, differences in sputum conversion rates and improvements in TB score. An ad hoc analysis looked at differences in the cytokine response and clinical recovery in groups stratified as ‘Optimal’, ‘Insufficient’ and ‘Deficient’ by vitamin D levels at enrolment.

### Analysis

Data was analysed by ‘intention-to-treat’ analysis. Outcome variables were reported by either their means or medians and with ranges or standard deviations. Statistical comparisons at 0 and 12 weeks were performed using Student’s *t*-test for continuous variables and Pearson Chi-squared tests for categorical variables. IFN-g responses pre- and post-treatment between baseline 25-(OH)hydroxy vitamin D levels and TB severity groups were compared using the Kruskal-Wallis non-parametric test. A two-sided *p* value of < 0.05 was considered significant. Data was analysed using SPSS version 17.0. An interim safety analysis looking for differences in mortality was carried out with the institutional research office at 3 months. No differences in mortality rates were identified between the two study groups.

## Results

### Description of the study

**Figure 1:**
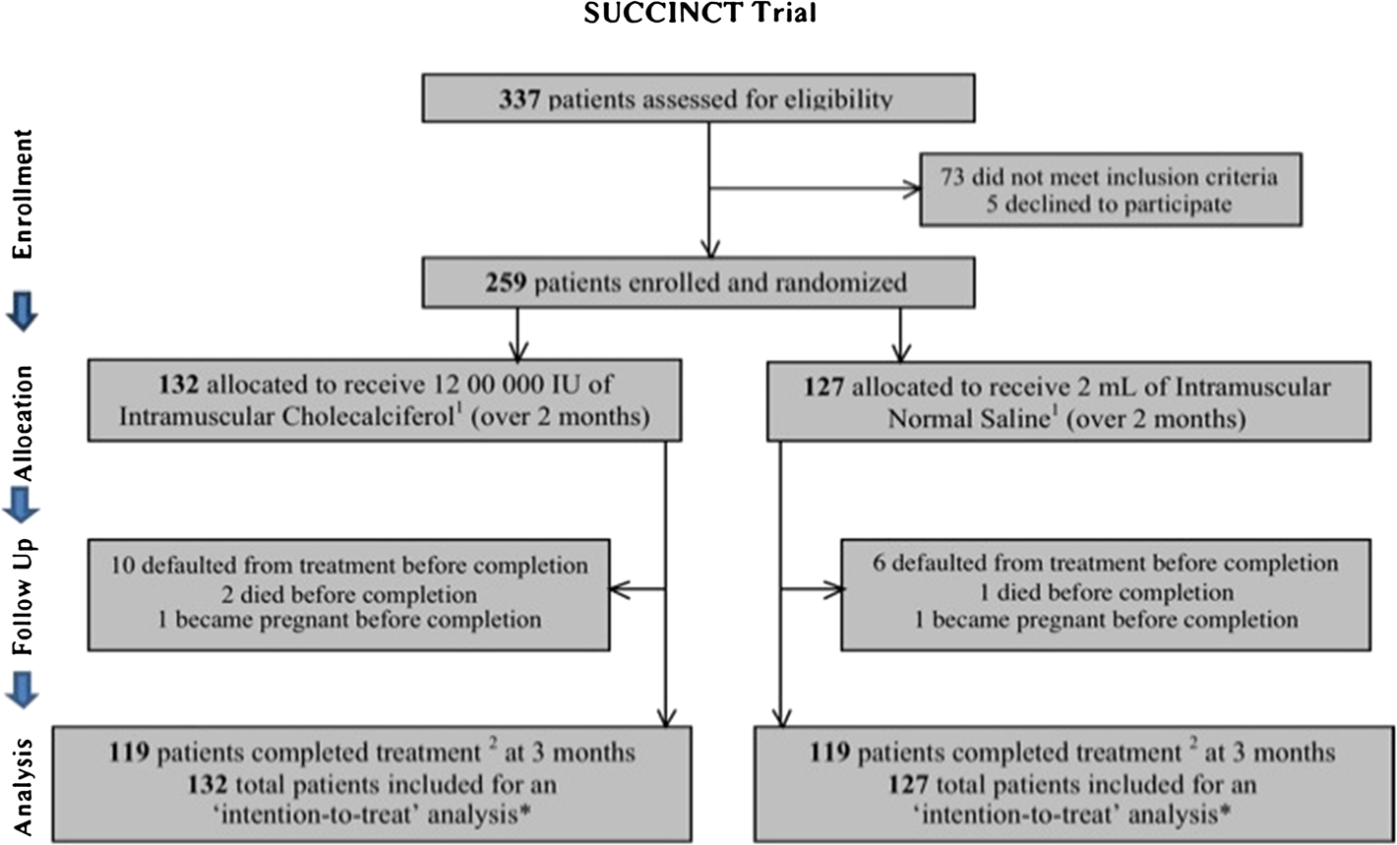
**Study Flowchart.** Study Drug (Cholecalciferol) and Placebo (Normal Saline) matched for colour and volume of contents. ^2^ ‘Completed treatment’; administration of 2 doses of study drug/placebo (over first 2 months), with follow up assessments complete at all visits over a total duration of 3 months. * Includes all patients who completed treatment at end of 3 months, as well as those who died during, or defaulted from, treatment before completion (indicated for each treatment arm). Flow Diagram adapted from the CONSORT 2010 Statement: updated guidelines for reporting parallel group randomized trials.

Three hundred and thirty seven patients were screened for eligibility; 259 patients were enrolled and randomized to the 2 treatment arms; 132 in the 25-hydroxyvitamin D supplementation arm and 127 in the placebo arm, as illustrated in Figure 1. The study was conducted from October 2009 to July 2010 with enrolment of the first participant in October 2009 and last participant in April 2010. One hundred and nineteen patients in each arm completed the study with a default/dropout rate of 8.1%. Three patients died during the study period; 2 in the 25-hydroxyvitamin D intervention arm and 1 in the placebo. Deaths in the vitamin D arm included 1 due to rapidly progressive respiratory failure of undetermined etiology within 2 weeks of inclusion and 1 in an automobile accident. One patient died of malaria in the placebo arm.

### Study findings

Baseline characteristics of the 2 study populations are shown in Table 1. The two arms did not differ significantly except for a higher number of patients with fever in the placebo arm, 49 v/s 68 (*p* 0.008). Mean age for the total population was 28 ± 13.6 years and 135 subjects (49.5%) had + 3 AFB on sputum smear microscopy. Mean 25-hydroxyvitamin D levels for the entire population were in the ‘Insufficient’ range; 21.3 ng/mL ± 9.78. 25 hydroxyvitamin D levels were noted to be lower in patients with greater severity of chest radiographic involvement at baseline [216 (83%) v/s 43 (16%), *p* 0.012], in females [67 (56.7%) v/s 49 (34.7%) had levels < 20 ng/mL, *p* 0.001] and in younger patients [*p* 0.004]. There were no significant associations between baseline 25-hydroxyvitamin D levels and sputum AFB load (*p* 0.77), or TB severity score (*p* 0.26). There was a trend towards greater association of haemoptysis with lower levels of vitamin D (*p* 0.049).

Changes in measured clinical variables after 12 weeks of antituberculous therapy are shown in Table 2. Changes in serum 25 hydroxyvitamin D levels in the two groups is shown in Additional file 1: Figure S1. After 12 weeks of antituberculous therapy, the 25-hydroxyvitamin D supplemented arm had a mean weight gain (kg) of; + 3.75 (3.16 – 4.34) v/s + 2.61 (95% CI 1.99 – 3.23) in the placebo arm (*p* 0.009) and in BMI; + 1.39 (95% CI 1.15 – 1.63) v/s + 0.95 (95% CI 0.71 – 1.19) in the placebo arm (*p* 0.01). Interpretation of chest radiographs at 12 weeks showed that the mean number of zones involved in the vitamin D arm were 1.35 v/s 1.82 zones in the placebo arm (*p* 0.004) (95% CI 0.15, 0.79). 106 (89.8%) patients in the vitamin D arm had 50% or more reduction in cavity size v/s 111 (94.8%) in the placebo arm (*p* 0.035).

**Table 2:** Changes in measured clinical variables from baseline to study completion

**Measured disease parameter(s)**	**Randomization**		***p*-value (95% CI)**
	**Drug intervention (n = 132)**	**Placebo intervention (n = 127)**	
**TB Severity (clinical assessment)**			
Mean Δ in TB score (points) ± SD, (95 CI)	- 3.19 ± 2.37, (−3.61, -2.75)	- 2.79 ± 2.44, (−3.23, -2.34)	0.198
Mean Δ in weight (kg), (95% CI)	+ 4.02, (3.18 – 4.86)	+ 2.61, (1.99 – 3.23)	0.007
Mean Δ in BMI, (95% CI)	+ 1.48, (1.17 – 1.78)	+ 0.96, (0.72 – 1.20)	0.008
Mean Δ in MUAC (cm), (95% CI)	+ 1.34, (0.74 – 1.64)	+ 0.97, (0.68 – 1.26)	0.079
**Chest X-Ray Involvement**			
Mean no. of zones^1^ involved ± SD	1.35 ± 1.13	1.82 ± 1.35	0.004
**Sputum Smear**			
Smear Conversion, no. (%) ^2^	108 (81.8)	103 (81.1)	0.39
**IFN-g levels (pg/ml) mean ± SD**			
Unstimulated levels	3.9 ±28.7	4.3 ± 45.2	0.325
ESAT6-stimulated*	387 ±920	206 ±665	0.077
MTBs-stimulated*	3092 ±1363	2987 ±1510	0.497

*MUAC* Mid Upper Arm Circumference; *BMI* Body Mass Index; 95 *CI* 95% Confidence Interval; *SD* Standard Deviation; s-25-(OH) D3 = serum 25-hydroxyvitamin D3.

^1^ Division of bilateral ling fields into a total of 6 zones (3 per each lung, ‘zone involvement’ = active parenchymal and cavitary disease, exclusive of old fibrotic scarring. ^2^ Includes all patients who were expectorating at baseline, and claimed ‘unable to expectorate’ at completion of treatment 12 weeks later. ESAT6 early secreted and T cell activated antigen-6 kDa; MTB_s_ Mycobacterium tuberculosis whole sonicate antigen.

*Denotes cytokine levels after subtraction of background from unstimulated cells.

Evaluation of sputum microscopy data for the study subjects showed that 134 (60%) patients were sputum smear negative by week 4 of therapy. Overall, no significant differences were observed in sputum smear conversion rates (Additional file 2: Figure S2) or TB scores at weeks 4 (*p* 0.18), 8 (*p* 0.89) and 12 (*p* 0.16) between the 2 study arms. On post hoc analysis, patients in the vitamin D arm and serum < 30 ng/mL (‘Insufficient’ and ‘Deficient’ groups) at enrolment had significantly greater improvements in TB severity scores compared to patients with normal baseline 25-hydroxyvitamin D levels; *p* 0.014. Additionally, the subgroup in the supplementation arm that had baseline ‘Insufficient’ levels showed a trend towards lower number of zones involved on chest radiograph (*p* 0.054) and higher sputum smear clearance (*p* 0.05) however, this difference was not statistically significant.

### Mycobacterial-antigen stimulated IFN-g responses in placebo and treatment groups

**Figure 2:**
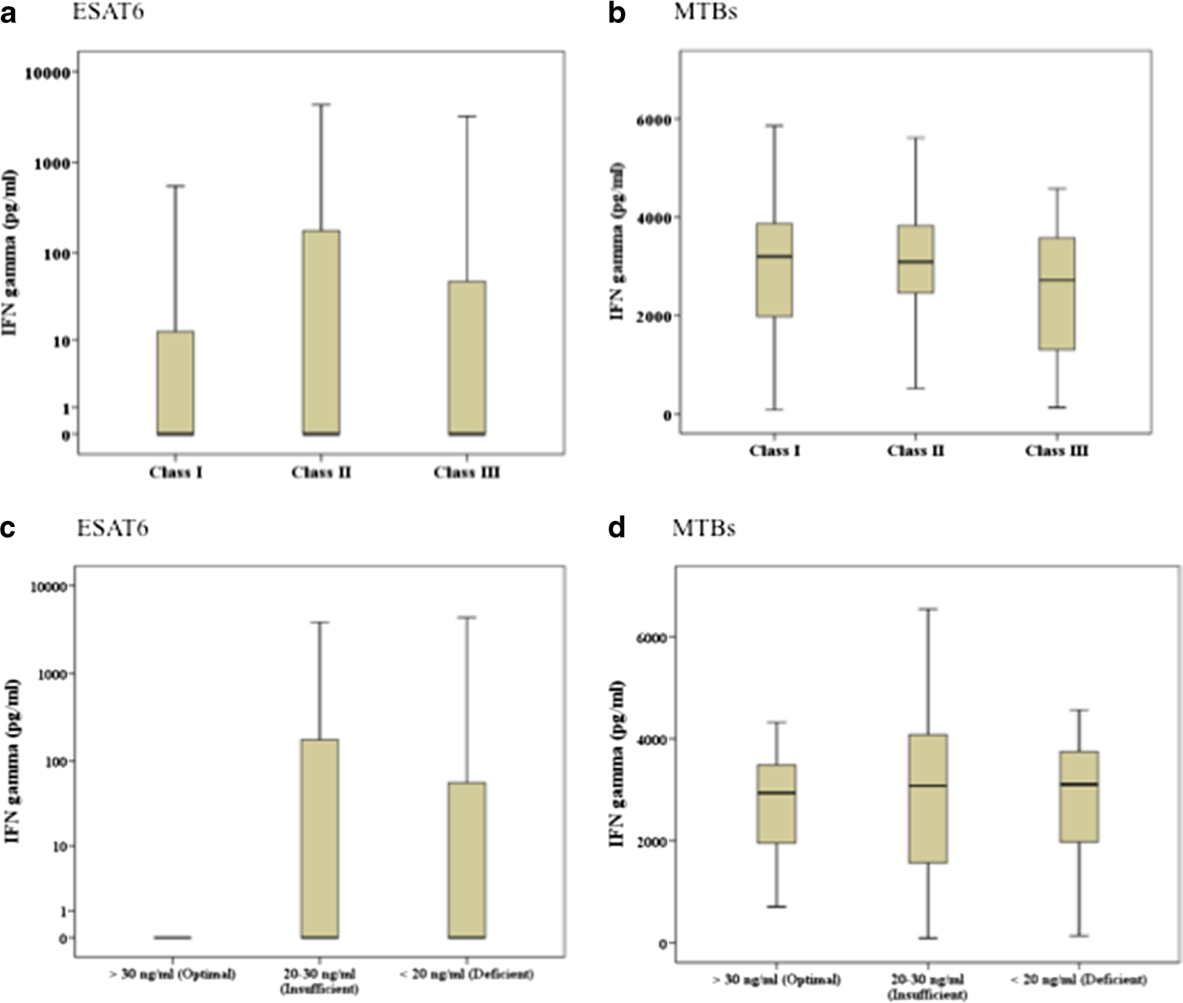
**Mycobacterial-antigen stimulated IFN-g responses in TB patients according to disease severity and circulating 25-hydroxyvitamin D levels.** Diluted whole blood cells were stimulated with ESAT6 and *M. tuberculosis* sonicate (MTBs) and IFN-g measured in cell supernatants after 6 days of culture. The graphs depict IFN-g responses in patients classified into severity according to their TB scores (**a-b**); Severity Class I (TB score 0 to 5), Class II (TB score 6 – 7) and Class III (TB score ≥ 8) in response to stimulation with (**a**) ESAT6 and (**b**) MTBs, or according to their vitamin D levels (**c-d**); Optimal (>30 nmol/ml), Insufficient (20–30 nmol/ml) or Deficient (< 20 nmol/ml) in response to stimulation with (**c**) ESAT6 and (**d**) MTBs. The box and whiskers plots depict cytokine responses in the 10th to 90th percentiles with the horizontal bar indicating the median levels of each group.

We first determined mycobacterial antigen-stimulated IFN-g responses at baseline in the placebo group to investigate possible effects in cytokine secretion profiles due to variations in disease severity in patients. We observed that ESAT6- induced IFN-g responses did not differ between patients classified according to their TB scores into Class I, II and III disease (p = 0.996, Kruskal-Wallis test, Figure 2A). Also, *M. tuberculosis* sonicate (MTBs) – induced IFN-g was similar between patients of with Class I, II and III TB (p = 0.257, Kruskal-Wallis test, Figure 2B).

As study subjects had variable circulating levels of 25-hydroxyvitamin D and vitamin D suppresses IFN- γ production in the host.^1^ we classified patients into those with Optimal, Insufficient or Deficient 25-hydroxyvitamin D levels at baseline and then determined mycobacterial antigen-stimulated IFN-g responses to see if there was any association between serum 25 hydroxyvitamin D levels and IFN-g responses in the placebo group. ESAT6 stimulated whole blood cell IFN-g secretion of groups with Optimal, Insufficient or Deficient 25-hydroxyvitamin D levels did not differ (p = 0.503, Kruskal-Wallis analysis), Figure 2C. MTBs-induced IFN-g secretion was also comparable between the different vitamin D groups (p = 0.608, Kruskal-Wallis analysis), Figure 2D.

### Increased MTBs-induced IFN-g responses with supplementation in those with Deficient 25-hydroxy vitamin D levels

**Figure 3:**
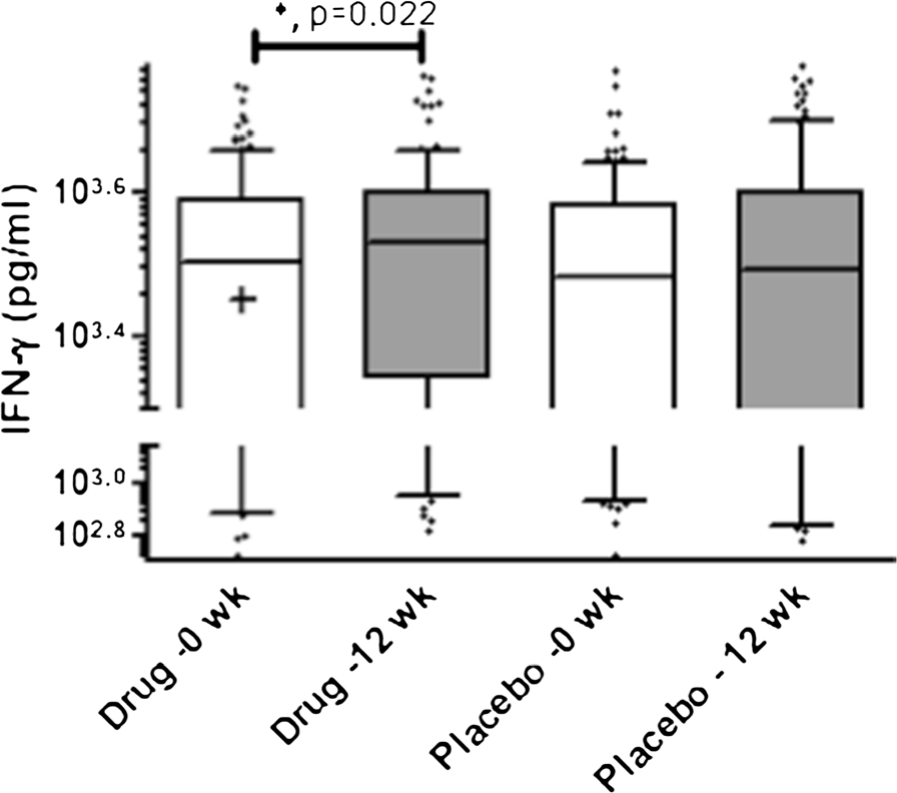
**MTBs-induced IFN-g secretion is increased in the Vitamin D treatment arm after 12 weeks of anti-tuberculous therapy.** Diluted whole blood cells were stimulated with *M. tuberculosis* sonicate (MTBs) and IFN-g measured in cell supernatants after 6 days of culture. The box and whiskers plots depict cytokine responses in the 10th to 90th percentiles with the horizontal bar indicating the median levels of each group. ‘*' Denotes values significantly different p < 0.05 between levels at 0 and 12 weeks respectively as determined by paired *t* test analysis.

We subsequently determined mycobacterial antigen-stimulated IFN-g responses prior to initiation of therapy (0 week) and after 12 weeks of therapy in both placebo and intervention arms of the study. It was observed that MTBs-induced IFN-g levels in the 25-hydroxyvitamin D intervention group were increased post-therapy (p = 0.022, Figure 3). However, no difference was observed in MTBs-induced IFN-g levels compared between 0 and 12 weeks in the placebo group. When ESAT6- stimulated IFN-g responses were considered no difference was observed between 0 and 12-week responses in either the placebo or intervention arms (Additional file 3: Table S1).

Further, we investigated factors which may play a role in determining the increase in MTBs- stimulated IFN-g responses in the intervention group post-therapy and further analysed this data after sub-classification of patients according to disease severity and also their baseline serum level of 25-hydroxyvitamin D levels.

### *M. tuberculosis* sonicate antigen-induced IFN-g responses are increased as a consequence of treatment in patients with more severe TB

**Figure 4:**
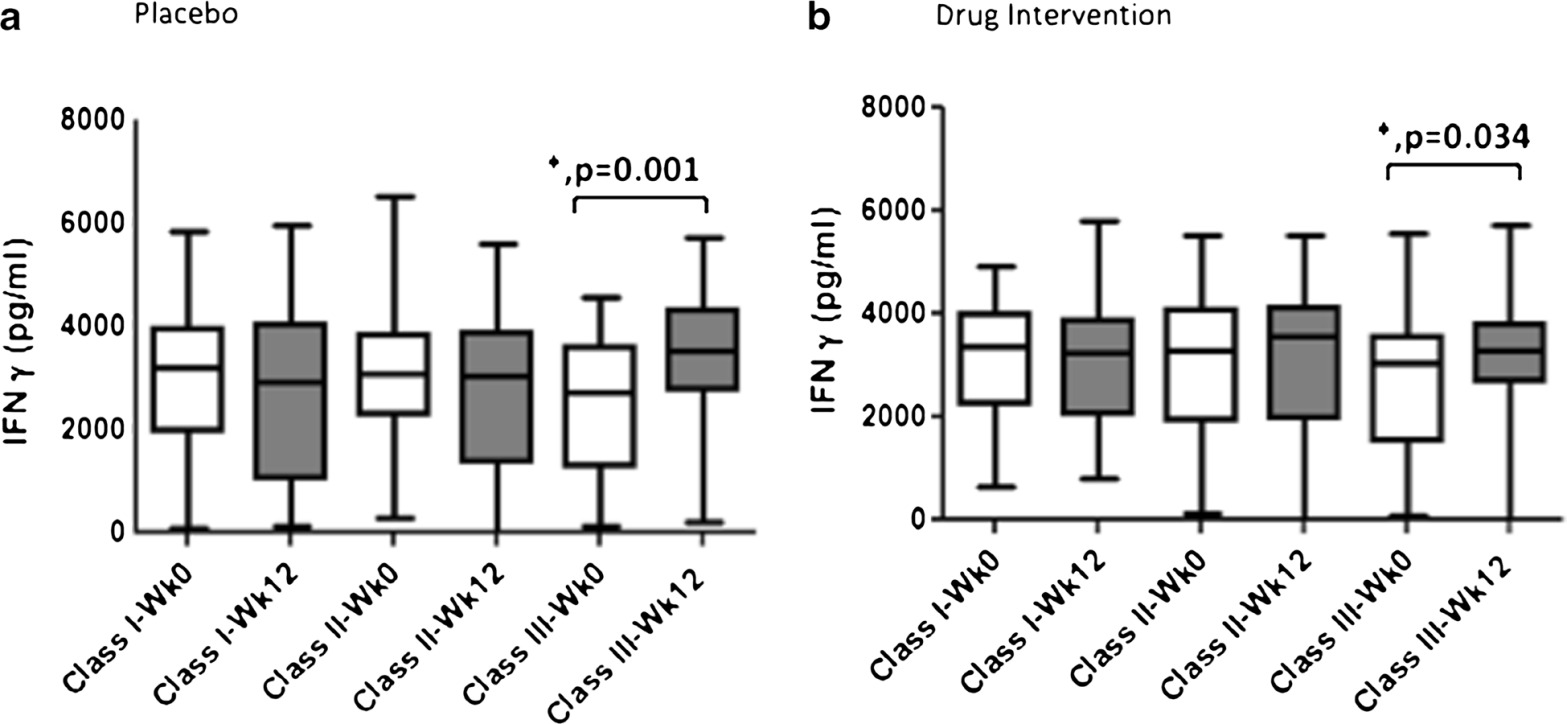
**MTBs- stimulated IFN-g levels are increased post-therapy in TB patients with deficient more severe TB.** Whole blood cells were stimulated with MTBs and IFN-g measured in cell supernatants and compared between patients classified into TB severity classes I, II and III. Responses at 0 and 12 weeks were compared in each group by paired *t* test analysis. The box and whiskers plots depict cytokine responses in the 10th to 90th percentiles with the horizontal bar indicating the median levels of each group. **a**, Placebo arm **b**, Drug intervention arm. ‘*' Denotes p <0.05 between levels at 0 and 12 weeks respectively.

It was observed that MTBs – induced IFN- γ responses significantly increased in patients with Class III disease after 12 weeks of anti-tuberculous therapy in both the placebo (p = 0.001) and 25-hydroxyvitamin D treatment arm (p = 0.034), Figure 4A-B. No such post-treatment increase was observed when ESAT6-induced IFN- γ responses in the study subjects were compared (Additional file 4: Table S2).

### Supplementation increases in *M. tuberculosis* sonicate induced IFN-g responses in patients with deficient 25-(OH) vitamin hydroxyvitamin D levels

**Figure 5:**
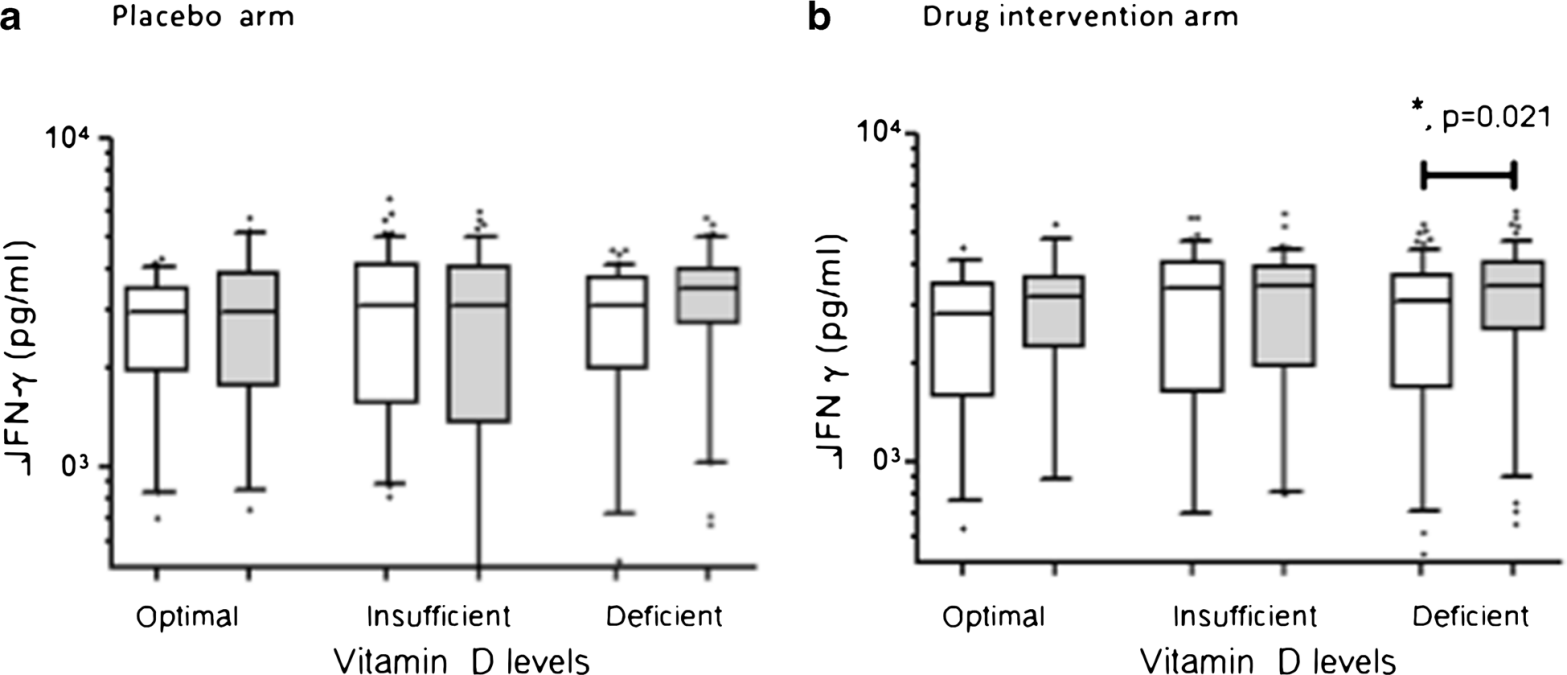
**MTBs- stimulated IFN-g levels are increased post-therapy in TB patients with deficient Vitamin D levels.** Whole blood cells were stimulated with MTBs and IFN-g measured in cell supernatants at 6 days post-stimulation. Vitamin D levels >30 ng/ml, Optimal; 20–30 ng/ml, Insufficient; <20 ng/nl, Deficient. **a**, Placebo arm **b**, Drug intervention arm. The box and whiskers plots depict cytokine responses in the 10th to 90th percentiles with the horizontal bar indicating the median levels of each group. ‘*' Denotes p <0.05 between levels at 0 and 12 weeks respectively as determined by paired *t* test analysis.

To investigate the effect of 25-(OH) vitamin D supplementation in patients with different serum 25-hydroxyvitamin D levels we compared subjects in both arms of the study classified into those with Optimal, Insufficient and Deficient 25-hydroxyvitamin D levels. Increase in MTBs-induced IFN-g secretion after therapy occurred only in subjects with Deficient 25-hydroxyvitamin D levels (p = 0.021, Figure 5B) in the drug intervention group but not in the placebo group, Figure 5A. ESAT6 induced – IFN- γ responses at 0 and 12 weeks did not differ in either the placebo or 25-hydroxyvitamin D treatment arms (Additional file 5: Table S3).

## Discussion

We have shown that therapeutic doses of Vitamin D (in the form of 2 doses of cholecalciferol at monthly intervals), given to patients with active pulmonary TB, can lead to proportionately greater weight gain and more rapid radiographic clearing of disease as compared to placebo.

It has recently been proposed that vitamin D accelerates resolution of host inflammatory responses and this may contribute to the improvement observed in vitamin D supplemented TB therapy [14]. Here we illustrate that vitamin D supplementation enhances mycobacterial-antigen induced IFN-g secretion in patients with Deficient 25-hydroxyvitamin D levels, thereby improving cell mediated immunity against *M. tuberculosis*.

25-hydroxyvitamin D is recognized as an important immune-modulator in TB. 1, 25 (OH) _2_ D_3_ binding with VDRs activates cathelicidin-mediated mycobacterial killing [1,2] whilst 25-hydroxyvitamin D deficiency increases the susceptibility and vulnerability to TB [3,4]. G interferon (IFN-γ) is a proinflammatory cytokine, which plays a critical role in resistance to MTB infection [15]. Infection with MTB induces T lymphocytes, natural killer cells and alveolar macrophages to express IFN-g and induces IFN-γ-driven monokines that regulate granuloma formation [16]. IFN-g responses have been shown to be depressed in patients with advanced forms of tuberculosis [17]. We observed a significant improvement in MTBs-induced IFN-g responses after 12 weeks of ATT in vitamin D ‘Deficient’ patients who received 25-hydroxyvitamin. This is the first vitamin D supplementation study where groups have been stratified into those with differing disease severity and also according to their baseline serum 25-hydroxyvitamin D levels.

Previous reports by Ulrichs et al. have shown that IFN-g producing cells against ESAT6 in tuberculosis patients increase post-tuberculosis therapy [18]. However, we were unable to demonstrate any difference in ESAT6-induced IFN- γ responses between patients prior to and post-treatment. This is in concordance with a recent report by Coussens et al. [14] that did not show any change in ESAT-6 induced IFN-g in patients post-antituberculous therapy. ESAT6 and culture filtrate protein 10 (CFP10) are both encoded by the region of difference 1 (RD1) present in *M. tuberculosis* and in virulent *M. bovis,* but absent from *M. bovis* BCG and environmental mycobacteria [19]. Antigen based interferon g release assays (IGRAs) have a varied sensitivity in endemic and non-endemic settings, attributable to exposure to environmental mycobacterial and *M. tuberculosis* and resulting T cell IFN-g responses [20]. *M. tuberculosis* whole sonicate (MTBs) contains cross reactive epitopes to *M. bovis* BCG vaccine strain and environmental mycobacteria and therefore, can induce potent cytokine responses from T cells, macrophages and other polymorphonuclear cells. MTBs induced responses have been useful in differentiating severity of disease in TB. Patients with advanced disease display decreased IFN-g responses [13]. We observed that after 12 weeks of ATT, the Class III severity groups had significant increases in MTBs-induced IFN-g responses. This increase in mycobacterial-antigen induced IFN-g responses represents host immunity in patients with advanced pulmonary disease, possibly leading to improved resolution of the disease. Further analysis of to determine the impact of host 25- hydroxyvitamin D levels on immune recovery revealed that in patients who received vitamin D supplementation, MTBs-induced IFN-g secretion was significantly increased after 12 week of anti-tuberculous therapy only in patients who had ‘Deficient’ 25-hydroxyvitamin D levels (< 20 ng/mL) at initiation of treatment whilst this immune recovery was absent for TB patients with ‘Deficient’ 25- hydroxyvitamin D levels in the placebo group. This data suggests a role for 25-hydroxyvitamin D supplementation in boosting host immunity particularly in those with deficient 25 hydroxyvitamin D levels. It is possible that only patients with ‘Deficient’ 25 hydroxyvitamin D levels showed an improvement perhaps due to a critical level of 25-hydroxyvitamin D required by the host for optimal activation of IFN-g secretion and because this limit was already reached in the other 25-hydroxyvitamin D groups where no enhancement of mycobacterial antigen-induced IFN-g response was observed.

Weight gain is routinely followed as an outpatient marker of clinical improvement in TB and we observed improvements in clinical status; i.e.; weight gain compared to the placebo group. This corresponds with the earliest reports of the benefits of vitamin D in TB patients published in 1848 [21] that describes disease arrest, weight gain and reduction in mortality in patients with TB treated with cod liver oil compared to standard therapy alone. More recently, Martineau et al. demonstrated that a single oral dose of 2.5 mg (100,000 IU) of ergocalciferol significantly reduced growth of mycobacteria [7]*.* A randomized, placebo controlled study on 67 Indonesian patients, by Nursyam et al. [22] reported that pulmonary TB patients given 420,000 IU of vitamin D over 6 weeks had significantly higher sputum conversion rates as compared to placebo (p 0.002). Martineau et al. [8] showed that 100,000 IUs of 25-hydroxyvitamin D_3_ supplementation significantly improved sputum conversion rates in patients with the T*aq1* 25-hydroxyvitamin D receptor polymorphism of the *tt* genotype. We speculate that this occurred due to broader effects of 25-hydroxyvitamin D on muscle, vascular and homeostasis [23-26].

We also found that the vitamin D treatment group had lesser disease (compared to placebo) by chest radiography after 12 weeks of therapy. This finding is consistent with a recent small placebo controlled trial on 24 children in Egypt that reported greater clinical and radiographic recovery after 1000 IUs of oral vitamin D supplementation [27] and a case report of an African –American patient with refractory, drug sensitive TB who improved only after being treated with 1,200,000 IU of ergoclaciferol [28].

However, two recently published large randomised, controlled trials by Martineau [8] and Wejse et al. [9] found no difference in clinical outcomes or mortality after 10 mg (400,000 IU) of 25-hydroxyvitamin D_3_ and 300,000 IUs of cholecalciferol or placebo were given to 146 pulmonary TB patients in London, United Kingdom and 365 TB patients in Guinea-Bissau. It maybe speculated that the differences in response to 25-hydroxyvitamin D seen between our study and Wejse’s could be due to variations in VDR polymorphisms, variability in 25-hydroxyvitamin D dosages or differing levels of baseline serum 25-hydroxyvitamin D levels.

We found 25-hydroxyvitamin D supplementation to be very safe, even in patients without deficiency. Only two patients in the vitamin D arm died during the study; one death was due to respiratory failure occurring within the initial 2 weeks of antituberculous therapy. It can be speculated whether this was a paradoxical response leading to acute lung injury.

We used a ‘higher’ dose than the 2 recent negative studies reported by Wejse and Martineau et al., but closer to therapeutic recommendations [29-31]. We speculate that this may account for the differences observed in the 2 groups at 12 weeks. We consider it a limitation of our study that we were unable to follow our patients to the end of treatment (6 months). It is possible that the benefits of 25-hydroxyvitamin D replacement may become more apparent between the groups with longer follow up.

Another limitation of our study is that we did not collect information on dietary intakes However the study groups were well matched at enrolment and represented a variety of socioeconomic and ethnic groups, which may reduce the possibility that one study arm was disproportionately better nourished.

## Conclusion

In summary, this study proposes that high dose vitamin D supplementation can lead to a more marked clinical and radiological recovery in all patients with pulmonary TB and boost host immune responses in patients deficient in 25-hydroxy vitamin D.

## Abbreviation

IFN- g: Interferon gamma; MTB: Mycobacterium tuberculosis; MTBs: Mycobacterium tuberculosis sonicate; TB: Tuberculosis; DOTS: Directly Observed Therapy; BMI: Body Mass Index; MUAC: Mid Upper Arm Circumference; VDR 25-hydroxyvitamin: Vitamin D receptors; TLR: Toll-like receptor; MMP: Mycobacterium-induced matrix metalloproteases; ATT: Anti-tuberculous therapy.

## Competing interests

The authors declare they have no competing interests.

## Authors’ contributions

NS and MA had full access to the data and take responsibility for the integrity of the data analysis. MA contributed to data collection, analysis and interpretation of data. FA contributed to study design, data collection, analysis and interpretation of data. ZH contributed to study design, data collection, analysis and interpretation of data. NR contributed to data collection. FM contributed to study design, analysis and interpretation of data. NS contributed to study conception and design, supervision of research, analysis and interpretation of data, drafting and critical revision of the manuscript for intellectual content, and statistical analysis. All authors read and approved the final manuscript.

## Pre-publication history

The pre-publication history for this paper can be accessed here:

http://www.biomedcentral.com/1471-2334/13/22/prepub

## Supplementary Material

Additional file 1 **Figure S1.** Serum 25-hydroxy Vitamin D levels in the 2 study groups over the course of the study. Click here for file

Additional file 2 **Figure S2.** Sputum Smear AFB Conversion rates in the 2 study groups002E. Click here for file

Additional file 3 **Table S1.** ESAT6- and MTBs-stimulated IFN-g responses in whole blood cells of TB patients. Data depicts IFN-g ♦secretion in unstimulated and antigen stimulated whole blood cells. ESAT6 early secreted and T cell activated antigen-6 kDa; MTBs, Mycobacterium tuberculosis whole sonicate antigen. Click here for file

Additional file 4 **Table S2.** ESAT6-induced IFN-g responses in patients with differing severity of TB. Patients with TB were divided into groups according to their TB scores; Severity Class I (TB score 0 to 5), Class II (TB score 6 – 7) and Class III (TB score ≥ 8). The data depicts IFN-g secretion in cellular supernatant of whole blood cells either unstimulated or after stimulation with ESAT6 (early secreted and T cell activated antigen-6 kDa). Values between groups were compared using determined by Kruskal-Wallis analysis. Click here for file

Additional file 5 **Table S3.** ESAT6-stimulated IFN-g responses in whole blood cells of TB patients with differing 25-hydroxyvitamin D levels. Data depicts ESAT6-induced IFN-g secretion in whole blood cells after subtraction of spontaneous secretion from unstimulated cells (was equivalent to 0 pg/mL) in each case. Values between groups were compared using determined by Kruskal-Wallis analysis whereby values p < 0.05 were considered significantly different. NS – not significant values. p < 0.05 were considered significantly different. Click here for file
