# Supplementary figures and images for: Vitamin D accelerates clinical recovery from tuberculosis: results of the SUCCINCT Study [Supplementary Cholecalciferol in recovery from tuberculosis]. A randomized, placebo-controlled, clinical trial of vitamin D supplementation in patients with pulmonary tuberculosis’
Source: BMC Infect Dis. 2013 Jan 19;13:22. doi: 10.1186/1471-2334-13-22 (PMC3556334; doi:10.1186/1471-2334-13-22)

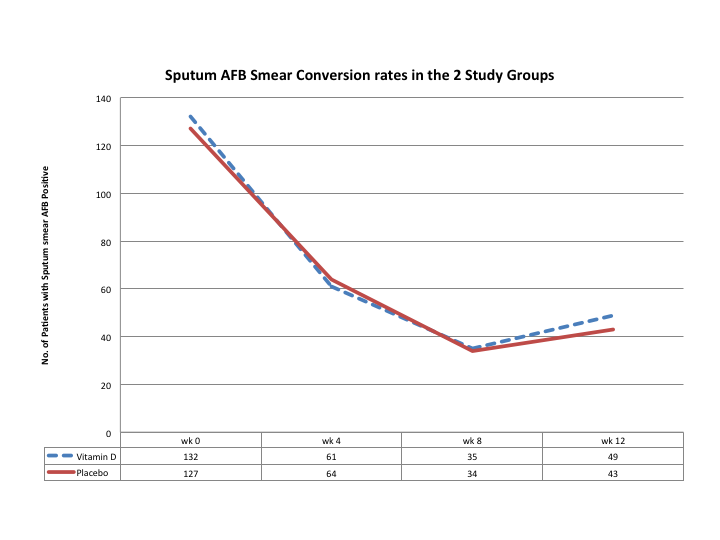

Supplement: Additional file 1 — Figure S1. Serum 25-hydroxy Vitamin D levels in the 2 study groups over the course of the study. [file 1471-2334-13-22-S1.tiff]

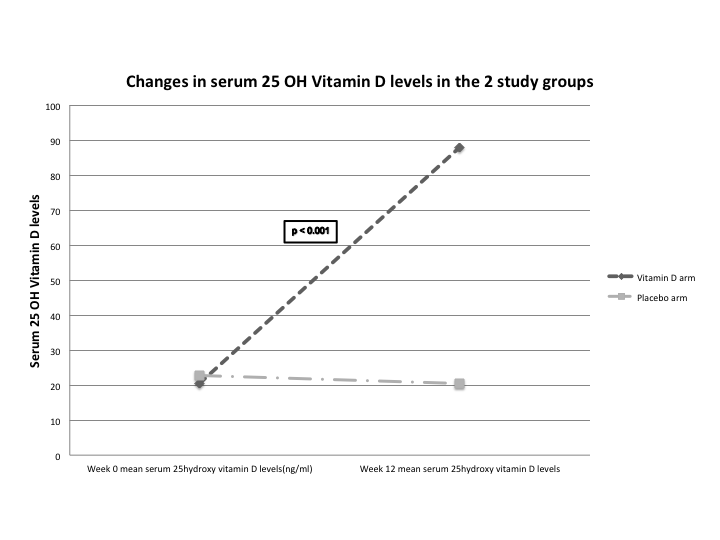

Supplement: Additional file 2 — Figure S2. Sputum Smear AFB Conversion rates in the 2 study groups002E. [file 1471-2334-13-22-S2.tiff]
