# Supplementary material for: Vitamin D accelerates clinical recovery from tuberculosis: results of the SUCCINCT Study [Supplementary Cholecalciferol in recovery from tuberculosis]. A randomized, placebo-controlled, clinical trial of vitamin D supplementation in patients with pulmonary tuberculosis’
Source: BMC Infect Dis. 2013 Jan 19;13:22. doi: 10.1186/1471-2334-13-22 (PMC3556334; doi:10.1186/1471-2334-13-22)
Supplement: Additional file 3 — Table S1. ESAT6- and MTBs-stimulated IFN-g responses in whole blood cells of TB patients. Data depicts IFN-g ♦secretion in unstimulated and antigen stimulated whole blood cells. ESAT6 early secreted and T cell activated antigen-6 kDa; MTBs, Mycobacterium tuberculosis whole sonicate antigen. [file 1471-2334-13-22-S3.doc]

**Supplementary Table 1. ESAT6-stimulated IFN-g responses prior to and after therapy in placebo and intervention groups**

**Unstimulated**

|  | Group | |  |
| --- | --- | --- | --- |
|  | Placebo | 25-hydroxyvitamin D |  |
|  | Median (25-75; IQR pg/ml) | Median (25-75; IQR pg/ml) | p-value |
| Week0 | 0 (0) | 0 (0) | NS |
| Week12 | 0 (0) | 0 (0) | NS |
| **ESAT6** |  |  |  |
|  | Group | |  |
|  | Placebo | 25-hydroxyvitamin D |  |
|  | Median (25-75; IQR pg/ml) | Median (25-75; IQR pg/ml) | p-value |
| Week0 | 0 (52.03) | 0 (151.88) | NS |
| Week12 | 0 (0) | 0 (119.18) | NS |

Data depicts IFN−g secretion in unstimulated and ESAT6-stimulated whole blood cells. Week 0 and week 12 values within groups were compared using the paired t- test analysis. NS – not statistically significantly; ‘*’ denotes values p<0.05 which are significantly different from each other.
