# Supplementary material for: Vitamin D accelerates clinical recovery from tuberculosis: results of the SUCCINCT Study [Supplementary Cholecalciferol in recovery from tuberculosis]. A randomized, placebo-controlled, clinical trial of vitamin D supplementation in patients with pulmonary tuberculosis’
Source: BMC Infect Dis. 2013 Jan 19;13:22. doi: 10.1186/1471-2334-13-22 (PMC3556334; doi:10.1186/1471-2334-13-22)
Supplement: Additional file 4 — Table S2. ESAT6-induced IFN-g responses in patients with differing severity of TB. Patients with TB were divided into groups according to their TB scores; Severity Class I (TB score 0 to 5), Class II (TB score 6 – 7) and Class III (TB score ≥ 8). The data depicts IFN-g secretion in cellular supernatant of whole blood cells either unstimulated or after stimulation with ESAT6 (early secreted and T cell activated antigen-6 kDa). Values between groups were compared using determined by Kruskal-Wallis analysis. [file 1471-2334-13-22-S4.doc]

**Supplementary Table 2. ESAT6-induced IFN−g responses in patients with differing severity of TB**

| **Unstimulated** | |  | |  | |  |
| --- | --- | --- | --- | --- | --- | --- |
|  | Week 0  Median (25-75; IQR) pg/ml | | Week 12  Median (25-75; IQR) pg/ml | | p-value | |
| Class I | 0 (0) | 0 (0) | | NS | |  |
| Class II | 0 (0) | 0 (0) | | NS | |  |
| Class III | 0 (0) | 0 (0) | | NS | |  |
| **ESAT6** |  |  | |  | |  |
|  | Week 0  Median (IQR) pg/ml | Week 12  Median (IQR) pg/ml | | p-value | |  |
| Class I | 0 (0-13.4) | 0 (0) | | NS | |  |
| Class II | 0 (0-201.7) | 0 (0) | | NS | |  |
| Class III | 0 (0-52.03) | 0 (0) | | NS | |  |

Patients with TB were divided into groups according to their TB scores; Severity Class I (TB score 0 to 5), Class II (TB score 6 – 7) and Class III (TB score ≥ 8).The data depicts IFN−g secretion in cellular supernatant of whole blood cells either unstimulated or after stimulation with ESAT6 (early secreted and T cell activated antigen-6kDa). Values between groups were compared using determined by Kruskal-Wallis analysis whereby values p< 0.05 were considered significantly different. NS – not significant
