# Supplementary material for: Vitamin D accelerates clinical recovery from tuberculosis: results of the SUCCINCT Study [Supplementary Cholecalciferol in recovery from tuberculosis]. A randomized, placebo-controlled, clinical trial of vitamin D supplementation in patients with pulmonary tuberculosis’
Source: BMC Infect Dis. 2013 Jan 19;13:22. doi: 10.1186/1471-2334-13-22 (PMC3556334; doi:10.1186/1471-2334-13-22)
Supplement: Additional file 5 — Table S3. ESAT6-stimulated IFN-g responses in whole blood cells of TB patients with differing 25-hydroxyvitamin D levels. Data depicts ESAT6-induced IFN-g secretion in whole blood cells after subtraction of spontaneous secretion from unstimulated cells (was equivalent to 0 pg/mL) in each case. Values between groups were compared using determined by Kruskal-Wallis analysis whereby values p < 0.05 were considered significantly different. NS – not significant values. p < 0.05 were considered significantly different. [file 1471-2334-13-22-S5.doc]

**Supplementary Table 3. ESAT6-stimulated IFN-g responses in whole blood cells of TB patients with differing 25-hydroxyvitamin D levels**

| **Placebo group** | |  |  |
| --- | --- | --- | --- |
| 25-hydroxyvitamin D | 0 week Median (25-75; IQR pg/ml) | 12 week Median (25-75; IQR pg/ml) | p-value |
| Deficient, > 30 ng/ml | 0 (0) | 0 (0) | NS |
| Insufficient, 20-30 ng/ml | 0 (0-181.6) | 0 (0) | NS |
| Deficient, < 20 ng/ml | 0 (0-66.9) | 0 (0-69.9) | NS |
| **Intervention group** | 0 week Median (25-75; IQR pg/ml) | 12 week Median (25-75; IQR pg/ml) | p-value |
| Deficient, > 30 ng/ml | 0 (0-25.1) | 0 (0-418.3) | NS |
| Insufficient, 20-30 ng/ml | 0 (0-914) | 0 (0) | NS |
| Deficient, < 20 ng/ml | 0 (0-6.1) | 0 (0-337) | NS |

Data depicts ESAT6-induced IFN−g secretion in whole blood cells after subtraction of spontaneous secretion from unstimulated cells (was equivalent to 0 pg/ml) in each case

Values between groups were compared using determined by Kruskal-Wallis analysis whereby values p< 0.05 were considered significantly different. NS – not significant
